# Oncofertility in Islam: The Malaysian Perspective

**DOI:** 10.3389/frph.2021.694990

**Published:** 2021-06-25

**Authors:** Mohd Faizal Ahmad, Nik Abdul Rahim Nik Abdul Ghani, Muhammad Azrai Abu, Abdul Kadir Abdul Karim, Mohamad Nasir Shafiee

**Affiliations:** ^1^Advanced Reproductive Centre, Faculty of Medicine, National University of Malaysia (UKM), Kuala Lumpur, Malaysia; ^2^Department of Obstetrics and Gynaecology, Faculty of Medicine, National University of Malaysia (UKM), Kuala Lumpur, Malaysia; ^3^Research Centre for Sharia, Faculty of Islamic Studies, National University of Malaysia (UKM), Bangi, Malaysia

**Keywords:** oncofertility, Muslim, cryopreservation, fertility preservation, Islamic

## Abstract

The implementation of oncofertility services is of great importance among cancer survivors of reproductive age to ensure a good quality of life. However, the uptake of this service among Muslim patients is very challenging because of inconclusive laws or evidence based on the Islamic perspective. Hence, we summarized the limited evidence available to consolidate current recommendations for oncofertility practices in the Muslim population in Malaysia. The available “fatwa” and “muzakarah” regarding reproductive procedures and gamete cryopreservation in Islam was searched from the recommendations of local and international bodies, including published and unpublished sources. The relevant information was then extracted according to the current understanding of dilemmas in oncofertility practices in Malaysia, tabulated, and consolidated. Most of the available “fatwa” has been revised by recent “muzakarah” to suit current oncofertility practices. Most Islamic organizations support the practice of oncofertility in Muslim hence indicates that oncofertility services are permissible. Therefore, proper recommendations and counseling are paramount to ensure understanding among Muslim patients.

## Introduction

Recent advances in cancer treatment worldwide have resulted in high survival rates. The use of new chemotherapeutic agents and radiotherapy modalities provides excellent cancer-cell control and curative outcomes among cancer patients, especially children and young individuals (1). However, the optimal balance of effective cancer treatments to achieve a good quality of life (QoL) following cancer survival is unknown. Most clinical providers overlook the post-cancer aspect of treatment, especially its reproductive sequalae (2). Fertility rates are remarkably affected by the selected treatment and, thus, may impair patients' QoL and lead to poor self-motivation. Most patients perceive cancer treatment to be a terminal solution and feel that subsequent life events are worthless, leading to poor compliance and treatment dropout (1–3). The emergence of the Oncofertility Consortium (OC) led by Prof. Theresa Woodruff in 2005 offered great hope and boosted the engagement of young cancer survivors with the hope that potential reproductive functions could be preserved despite cancer treatment (4). The benefits of the OC are mainly manifested by advancements in reproductive cryobiology, including gamete cryopreservation (sperm, oocytes), embryo cryopreservation, and tissue cryopreservation (testicular and ovarian tissue), all of which are offered by over 300 centers around the world (5). Despite the implementation of the OC, however, its acceptability varies, depending on a country's culture and religious beliefs, especially in Southeast Asia, which includes Malaysia (5, 6).

Malaysia, with its population of roughly 32.73 million, is the 43rd most populated country in the world and ranks third among the countries with the largest populations in Southeast Asia. Malaysian citizens can be divided into various local ethnicities, and bumiputras make up at least 67.4% of the population. The largest ethnic group in Malaysia are Malays, which, according to the constitution, are all Muslims. The bumiputra status is also accorded to the non-Malay indigenous groups of Borneo island: which includes Dayaks (Iban, Bidayuh, Orang Ulu), Kadazan-Dusun, Melanau, Bajau and others. Approximately 24.6% of the Malaysian population is made up of Malaysian Chinese, while 7.3% is made up of Malaysian Indians (7). Islam is the country's best-established religion, but the constitution grants freedom of religion to non-Muslims. However, modern laws and medical outlooks, including reproductive practices, align with the Islamic perspective (8, 9).

In previous years, fertility preservation in cancer cases was managed on an *ad hoc* basis without a dedicated referral center in Malaysia. The concept of oncofertility became popular in Malaysia in 2018, 2 years after the establishment of the Asian Society of Fertility Preservation, which aimed to facilitate and implement oncofertility services around Asia (6). Subsequently, the Ministry of Health launched the first National Referral Oncofertility Center on August 26, 2020 to establish a leading oncofertility referral center with a nationwide reach (6, 10). However, the acceptability of these efforts varies, especially among single Muslim patients. Doubts regarding gamete and tissue cryopreservation among cancer groups have remained given uncertainties in this practice from an Islamic perspective; thus, it should be dissected according to the Islamic perspective (11). This issue has led to the low uptake of OC services. Leaders thus believe that a proper Malaysian Islamic consensus/national “fatwa” is necessary to ensure that this service achieves its target. Therefore, in this work, we provide a review of the acceptability of different oncofertility practices based on the Islamic perspective to alleviate remaining concerns.

## Materials and Methods

A review was done on Islamic literature to determine the degree to which reproductive practice guidelines and Islamic scholarly articles reflect the need for and importance of incorporating cryopreservation as oncofertility treatment into Islamic preferences. The review of the available “fatwa” and “muzakarah” was also done regarding reproductive procedures and gamete cryopreservation in Islam from local and international bodies' recommendations, including published and unpublished sources. A relevant evidence established from 1981 until 2020 was included in this review. The literature portrays the changes of the Islamic landscape of cryopreservation rules from strictly prohibited to accepted based on available “fatwa” and recent “muzakarah.” The included literature was summarized in Table 1.

**Table 1:** The summary of law/recommendation chronologically from Islamic religious bodies.

**Law/recommendation**	**Year**	**Source**
The semen banking is consider illegal in Islam.	1981	*Muzakarah Fatwa* Committee National Council for Islamic Religious Affairs Malaysia
The sperm cryopreservation is considered illegal because it is likely to cause mixing of “*nasab*”, leading to business or commercialization.	2010	General Iftaa' Department, The Hashemite Kingdom of Jordan—available online; https://www.aliftaa.jo/Question2.aspx?QuestionId=675#.X-IQc9gzbIU
The sperm cryopreservation is forbidden because it causes a mixture of “*nasab*” and *tala'ub* (being the game material of those who want to profit)	2012	Online fatwa website (islamqa)—available online; https://islamqa.info/ar/answers/177178/ 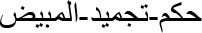
The oocyte cryopreservation is necessary for cancer patients (resolution no 248/ 17/2017) based on two basic methods: 1. The original ruling of treating is consider as “should” 2. The preserve oocytes should not be mixed and the “akad” of marriage should be respected. The fertilization must be done ONLY with valid marital bonding. This “*wasilah*” is allowed because there is a wish (requirement) whether oocyte can be taken before the marriage or within the marriage period.	2017	General Iftaa' Department, The Hashemite Kingdom of Jordan—available online; https://www.aliftaa.jo/Decision.aspx?DecisionId=550#.YBQf6nPivIU
In the fatwa: 373351 The sperm banking/cryopreservation is forbidden because it believed that the sperm won't remain in the sperm bank there is a possibility of a mixture of “*nasab*”	2018	Online fatwa website (islamweb.net) - available online; https://www.islamweb.net/ar/fatwa/373351/ 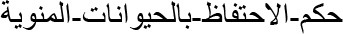
The gametes cryopreservation “should” be done on several conditions: 1. The process is done between husband and wife (legally married) 2. The care is optimum to prevent mixture of gametes 3. The cryopreserve cannot be used for others (third party) 4. There will be no adverse clinical outcome for the fetus in future	2019	Dar al-Ifta' al-Misriyyah- available online; https://www.dar-alifta.org/AR/Viewstatement.aspx?sec=mediaandID=6683
The cryopreservation of oocytes and sperm is must for cancer patients prior to chemotherapy or radiotherapy on several conditions: 1. The masturbation for men purposely for sperm cryopreservation is allow before married provided the treatment will lead to risk for his future fertility. 2. The baby born after married from the sperm or oocytes cryopreservation (done before married) is consider valid for the couple provided the baby born after 6 months after they married. 3. The health facilities able to be responsible to ensure no mixing or contamination of the gametes. 4. The health facilities able to ensure that only good quality of gametes is used to reduce complication in future. 5. The health facilities able to be honest and trustable in handling the gametes cryopreservation. 6. If the patients pass away after cryopreservation, the gametes MUST be discard immediately.	2020	Al-Bakri DDZBM. “*Muzakarah” “Syarak”* Law No 122; for Oocytes and Sperm Cryopreservation Bank among Single (Unmarried) Cancer Patient. Islamic Mufti Office of Federal Territory of Kuala Lumpur 2020 (unpublished)

## Islam in Malaysia

**Figure 1:**
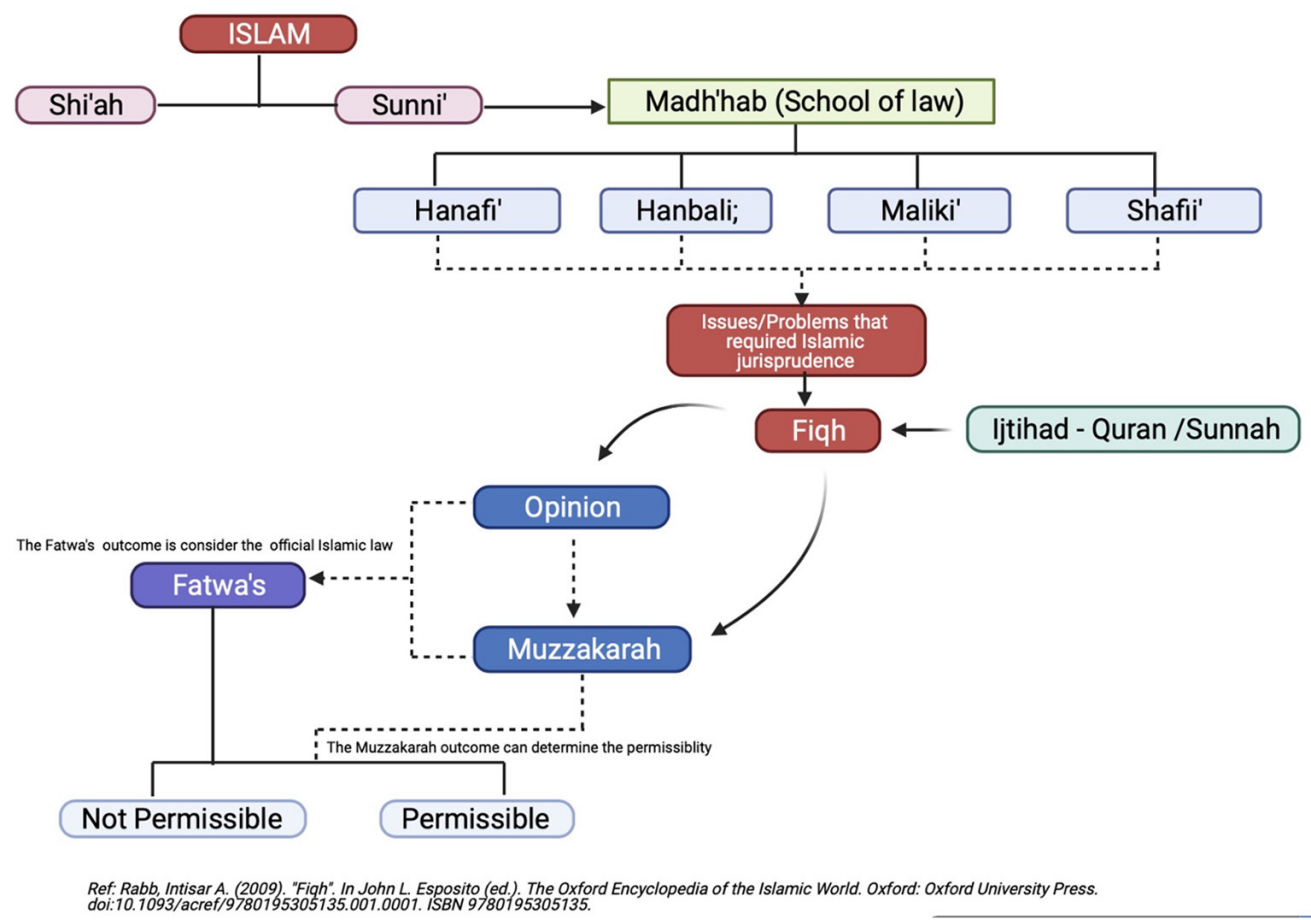
The flow of Islamic jurisprudence.

Islam is known as the official religion in Malaysia and, thus, blended into the general lifestyle of the country's citizens. The tenets of Islam influence all human activities to consolidate spiritual and material considerations in the individual, social, economic, political, and legal contexts (8, 12). The Malaysian Islamic core follows the general rules regarding practices and law, including blood sharing and family issues, as indicated in the Quran. As Islam affirms the importance of marriage, family formation, and procreation, any medical or scientific opinion concerning these topics is considered sensitive and complex as it is diverse according to two arms: shi'ah and sunni (11, 13). In general, Malaysian Muslims follow Sunni beliefs, which form the basis of 90% of all Muslim beliefs worldwide (Figure 1). The life and daily practices of Sunni believers originate from the “fatwa” (religious opinions/rulings) issued by the relevant religious bodies (14).

### Islam and the Current Practice of Reproductive Services in Malaysia

The fatwa regarding birth, death, marital issues and divorce, and assisted reproductive treatment (ART) is currently practiced in Malaysia following other Muslim-based countries (9, 11, 12, 14, 15). The core fatwa on ART practices among Muslims was established in 1980 by the Al-Azhar Religious Institution, which is based in Cairo, Egypt, and reviewed in 1984 by the Islamic Fiqh Council, which is based in Mecca, Saudi Arabia (16, 17). This fatwa has also led to the formation of fundamental guidelines from the Organization of Islamic Medicine in Kuwait (1991) and the Islamic Educational, Scientific, and Cultural Organization in Morocco (2002) to strengthen current practices related to ART among the Muslim population (17, 18). No written policy of reproductive law is available in Malaysia; thus, the majority of the practices implemented are dependent on the beliefs of the center and practitioner. However, most centers follow Muslim regulations as the basis of their practice. Currently, all forms of ART among Malaysian Muslims are allowed except for surrogacy and gamete donation, similar to the reproductive practices of other Muslim countries (15, 19). Gametes (sperm and oocytes) must be obtained from a husband and his wife. The embryo may be placed into the wife's uterus only under a valid marital contract, and no third-party involvement is allowed (16, 18, 20). Cryopreservation of sperms, oocytes, and embryos is also allowed as long as a couple remains married, and embryo transfer is done under a valid marital contract. The use of embryos as research materials is allowed, depending on the local or international Islamic consensus, as long as the embryo is aged <120 days given the belief that no “soul” enters the fetus before this period (12, 14, 21). Fetal reduction is also allowed if the pregnancy presents great risk to the mother or if more than two embryos are transferred under the context of achieving a viable pregnancy given low pregnancy potential (e.g., in case of recurrent implantation failure or advanced maternal age) (14). Pre-implantation Genetic Disease Testing (PGD) among Malaysian Muslims is currently allowed because it is considered a better option than the prenatal diagnosis and subsequent abortion of babies with potential genetic diseases; however, it is not allowed for sex preselection. While the fatwa by Al-Azhar allows PGD for sex preselection for family balancing (14, 21), it is not actually adopted and practiced in Malaysia. Surrogacy is practice among Shi'ah muslim but prohibited among Sunni Muslims (11). This is because procreation is only allowed within a legally binding marriage more for moralistic for his faith and progeny and ensure legitimate children (9, 15, 16, 22, 23). Without marital bonds, the relations of “nafkah” (daily expenses), “hadhanah” (custody), and “nas” (inheritance) could become a complex issue leading to chaos when rights are determined under Muslim law (17, 20, 24). Therefore, in Malaysia, surrogacy is not allowed among Muslims in general.

### Oncofertility Services in Malaysia: The Islamic Prospective

The implementation of oncofertility services presents an important impact to Malaysian reproductive services, especially from the Muslim perspective. Sperm freezing/banking, oocyte cryopreservation/banking, and embryo/tissue cryopreservation/banking are necessary to initiate oncofertility services in Malaysia. Therefore, a proper discussion of these matters is vital. The issue of sperm and oocyte cryopreservation was not discussed by classical Muslim scholars. Nevertheless, a few contemporary “fiqh” studies on rulings related to *in vitro* fertilization have been conducted. These studies generally reveal that sperm cryopreservation is prohibited in Islam because of the possibility of mixed progeny and commercialization of the practice. Therefore, it should be avoided according to the principle of “ihtiyat” (precaution) (17, 22, 25). Moreover, the available Islamic fatwas generally prohibit sperm and oocyte banking because unconscionable sperm and oocytes remain in the bank and are concerned about the possibility of mixing “nasab”; inheritance leads to chaos Muslim concern (16, 22, 26). Another concern is that the creation of these banks could lead to “tala'ub” (manipulation of the bank for profit by opportunistic groups) (17, 25, 26).

## Discussion

The fatwa from the “Muzakarah” (Religious issue meeting—National Fatwa Council Committee for Islamic Religious Affairs, Malaysia) on this issue considers the concepts of embryo cryopreservation and sperm banking. For example, the 1st “Muzakarah” Fatwa Committee National Council for Islamic Religious Affairs, Malaysia, which convened on January 28–29, 1981, focused on sperm banking (27).

The outputs of this meeting are as follows:

I. The initiation of sperm banks is illegal in Islam.
II. If a sperm bank is already in existence, the government shall act to remove it.
III. Insemination of persons made to humankind is illegal unless sperm from a husband is found to be “muharam” (legally married); in this case, insemination is approved by “syarak” (permitted by Islam).
IV. The involvement of specialist doctors or any party in sperm banks is illegal.

However, the above fatwas are superficial and require further evaluation and revision, especially in the context of oncofertility services and treatment. If an unmarried cancer patient undergoes chemotherapy or radiotherapy, which could potentially affect their fertility, cryopreservation may be deemed justified. If a patient wishes to keep their sperm, oocytes, or ovarian tissue and use the same in the future after healing and marriage, the prohibition of this act should be revised. Hence, several international fatwas consolidating recommendations regarding cryopreservation banking for oncofertility cases, such as the General Fatwa Department; Jordan, 2017 and fatwa Dar al-Ifta'; Egypt, 2019, have been published. Both of these fatwas recommend the use of cryopreservation banks strictly for cancer patients aiming to achieve fertilization with a partner to whom they are legally married if cryopreservation is conducted prior to the marriage (28, 29). The fatwas also allow the use of cryopreserved materials only by the married couple to obtain a healthy fetus following cancer treatment; these materials cannot be donated. The latest unpublished “Muzakarah” of the Islamic Religious Office in the Federal Territories of Kuala Lumpur follows the recommendation of Dar al-Ifta' al-Misriyyah; 2021- as such, this “muzakarah” is the basis of oncofertility services provided in Malaysia (16, 26, 30).

Because sperm, oocyte, and ovarian tissue cryopreservation is considered vital for cancer patients, the current Islamic rules or fatwa should be assessed from various aspects related to cancer care. The “syarak” often considers the intentions of “mukallaf” (the ultimate aim of a person's actions; in this case, oncofertility treatment). “Syarak” also considers “maslahah” (good/positive) and “mafsadah” (negative/disadvantage). Because the issue of gamete and tissue cryopreservation revolve around “maslahah” and “mafsadah,” each ruling varies accordingly (25). Izz al-Din explained that the available medical knowledge is in line with the “syarak” to achieve good and prevent harm (17). According to Al-Baz, the determination of the rules of oncofertility cryopreservation banks should be based on two fundamentals: (1) the balance between the “maslahah” and “mafsadah” of gamete and tissue cryopreservation and (2) factors supporting the act of cryopreservation of gametes and ovarian tissues (25). Previous fatwas banning sperm and oocyte banks were mainly based on strong concerns, such as the possibility of mixing “nasab” and profit-making by opportunistic groups. In the context of Shariah, “nasab” mixing concerns are included in the objectives of “hifz al-nasl” (preservation of progeny) (26). However, sperm, oocyte, and ovarian tissue cryopreservation for cancer patients also intends to preserve progeny because the reproductive organs of these patients are exposed to damage risk during cancer treatment. In this case, oncofertility treatment is considered in line with “hifz al-nasl” and supported by “syarak” (22). Therefore, oncofertility treatment may be considered an appropriate treatment strategy and implemented to ensure a better QoL for cancer patients.

To date, no written regulation of practices regarding oncofertility are available in Muslim-dominated countries, including Malaysia. Thus, our review is based on the recommendations of the related religious bodies and our understanding of the existing international fatwas and “muzakarah” (13, 26, 29, 30). Hence, special fatwas related to gamete and ovarian tissue cryopreservation in cancer cases, especially single patients, should be decided by an authoritative fatwa body, including that in Malaysia. Fatwas should also decide on “nasab” issues and all related matters to ensure a better understanding and good implementation of oncofertility services among Muslim patients.

## Conclusion

Our review indicated that gametes cryopreservation as an oncofertility treatment is permissible in Muslims as it is a fundamental option for cancer patients of reproductive age. Therefore, it should be made available to all patients, regardless of their religion. Our findings also provide a good platform for oncofertility treatment among Muslim patients dealing with its related issues. Otherwise, consistent recommendations from international and local fatwas are paramount to ensure general acceptability among Muslim patients worldwide, including Malaysia.

## Author Contributions

MFA and NARNAG: conceptualization and data curation. MAA, AKAK, and MNS: formal analysis. MFA, NARNAG, and MNS: methodology. MFA, NARNAG, AKAK, and MAA: project administration. NARNAG and MNS: supervision. MFA, NARNAG, MAA, AKAK, and MNS: writing—original draft, review and editing. All authors have read and agreed to the published version of the manuscript.

## Conflict of Interest

The authors declare that the research was conducted in the absence of any commercial or financial relationships that could be construed as a potential conflict of interest.
